# Insect Behavioral Change and the Potential Contributions of Neuroinflammation—A Call for Future Research

**DOI:** 10.3390/genes12040465

**Published:** 2021-03-24

**Authors:** Colleen A. Mangold, David P. Hughes

**Affiliations:** 1 Department of Entomology, College of Agricultural Sciences, Pennsylvania State University, University Park, State College, PA 16802, USA; cav154@psu.edu; 2 Center for Infectious Disease Dynamics, Huck Institutes of the Life Sciences, Pennsylvania State University, University Park, State College, PA 16802, USA; 3 Department of Biology, Eberly College of Science, Pennsylvania State University, University Park, State College, PA 16802, USA

**Keywords:** behavioral manipulation, glia, insect innate immunity

## Abstract

Many organisms are able to elicit behavioral change in other organisms. Examples include different microbes (e.g., viruses and fungi), parasites (e.g., hairworms and trematodes), and parasitoid wasps. In most cases, the mechanisms underlying host behavioral change remain relatively unclear. There is a growing body of literature linking alterations in immune signaling with neuron health, communication, and function; however, there is a paucity of data detailing the effects of altered neuroimmune signaling on insect neuron function and how glial cells may contribute toward neuron dysregulation. It is important to consider the potential impacts of altered neuroimmune communication on host behavior and reflect on its potential role as an important tool in the “neuro-engineer” toolkit. In this review, we examine what is known about the relationships between the insect immune and nervous systems. We highlight organisms that are able to influence insect behavior and discuss possible mechanisms of behavioral manipulation, including potentially dysregulated neuroimmune communication. We close by identifying opportunities for integrating research in insect innate immunity, glial cell physiology, and neurobiology in the investigation of behavioral manipulation.

## 1. Introduction

There are many organisms in nature that are able to impact the behavior of other organisms. These organisms provide outstanding platforms with which to study the co-evolution of specific host–parasite/parasitoid interactions and the underlying mechanisms that control certain animal behaviors. The most striking examples of behavioral change occur in invertebrates. Some affected behaviors include alterations in reproductive behavior, where and how hosts die, and overall changes in movement and locomotion. In many cases for microbial manipulators, their genomes have evolved to encode for specific proteins that directly or indirectly alter their host’s behavior [1,2,3,4,5,6,7,8].

It has been hypothesized that an organism is able to influence the behavior of another organism by one of three major mechanisms, as described in [9]: (1) altered neuroimmune communication, (2) active secretion of neuromodulators/changes in host synthesis of neuromodulators, and/or (3) altered host gene and/or protein expression. It is also likely that more than one of these mechanisms is used by behavioral manipulators to impact host behavioral change. Beautiful work has been done demonstrating the wide variety of parasite-specific factors and altered host cell signaling that are believed to contribute to the induction of host behavioral change [1,10,11,12,13,14,15,16,17,18,19,20]. However, less is known about how alterations in host immune signaling and immune function may also impact behavior in these systems. It is clear that in mammals, neuroimmune communication plays key roles in neuron development, synapse stability, and neuron function [21]. In many cases, glial cells (e.g., astrocytes and microglia) act upon neurons and regulate functions such as synaptic pruning, transmission, and plasticity [22,23]. Many of these actions may be regulated by immune signaling, including via the expression of different cytokines, major histocompatibility complex class I and II signaling, and complement pathway signaling [24,25,26,27]. Dysregulation of these signaling pathways may result in neuron dysfunction and degeneration [28]. Whether insect-specific immune factors have similar impacts on neuron function and synapse stability is less well known. Additionally, insect-specific glia–neuron communication mechanisms, glial cell function, and the potential consequences of aberrant glial cell activity on neurons in insects all remain relatively unclear. Increasing evidence in *Drosophila* models suggests similarity between insect and mammalian glial cell function and in the contributions of glial cell dysregulation in the manifestation of disease [29,30,31,32,33,34,35]. Behavioral manipulators may serve as outstanding model systems in which to study these processes in insects further. 

In this review, we will provide a brief overview of the insect immune system and then detail specific examples of microbes, parasites, and parasitoids that are able to induce behavioral change in the hosts that they infect. We describe the different behavioral phenotypes that they induce, the known mechanisms underlying host behavioral change, and the possible contributions of immune dysregulation toward altered host behavior in these systems. We also discuss parasite-mutualistic viruses, the potential immune-specific impacts of these viruses on host behavior during infection, and the importance of investigating these types of associations in behavioral manipulation. We then detail neuroinflammation in insects and the implications of immune signaling and glial cell signaling on neuron function. We close by discussing future avenues of research that can aim to elucidate the impacts of neuroimmune communication on insect behavior and the importance of investigating insect glial cell biology not only in the context of behavioral manipulation but also insect neurobiology

## 2. Insect Immunity—An Overview

The evolution of an effective immune system has made insects some of the most successful organisms on earth [36]. On a daily basis, insects continuously encounter pathogens including viruses, bacteria, fungi, and a variety of different parasites. In response, insects have evolved many different approaches to reduce infection probability and/or lethal infection. For example, insects possess a hard, outer cuticle layer that serves as a physical barrier to infection [37,38]. Additionally, some insects have evolved social immunity behaviors (or “collective immune defenses” against different pathogens [39]), which provide protection against disease. Behaviors within, but not exclusive to, eusocial insect colonies (e.g., ants and honey bees), including grooming interactions and physical removal of infected or dead individuals from the colony, help protect colonies from disease spread and potential colony collapse [37,39].

**Figure 1:**
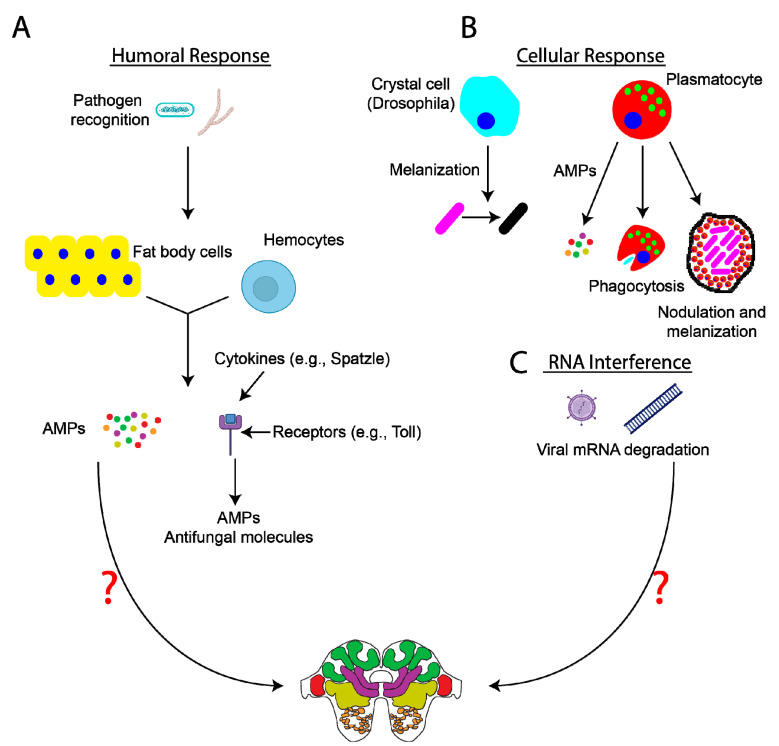
Insect immune responses and potential impacts on the nervous system. Insects utilize many different methods to fight off infection, which are reviewed here [37,40]. Briefly, (**A**) the humoral immune response involves the release of antimicrobial peptides (AMPs) from fat body cells and hemocytes into the hemolymph. AMPs aid in the targeting and destruction of different pathogens (e.g., bacteria and fungi). Intracellular signaling events (e.g., Spätzle/Toll, immune deficiency (IMD), and JAK–STAT signaling) are activated, leading to the synthesis of AMPs and the induction of different immune responses. Recent data have indicated the presence of immune priming in insects, making the insect immune system more adept at fighting off repeat infections than previously believed [43,46]. (**B**) Cellular responses to infection include, but are not limited to, the induction of processes such as melanization and nodulation, which effectively isolate and neutralize pathogens, and pathogen phagocytosis. Lastly, RNA interference (**C**) can mediate the degradation of viral genetic material. At present, it remains relatively unclear specifically how these processes (e.g., release of AMPs, alterations in intracellular signaling, phagocytosis, etc.) impact neural and glial cell function and host behavior (as indicated by the question marks). Images created with BioRender.com (accessed on January 15, 2021).

While the presence of a cuticle and the manifestation of certain social immunity behaviors help protect insects from infection and/or disease spread, insects also possess a highly efficient immune system, which aids in the rapid recognition and destruction of invading pathogens. The exact mechanisms of insect immunity will not be discussed in depth here but are nicely reviewed in [36,37,38,40]. In brief, the insect immune system is split into humoral (Figure 1A) and cellular (Figure 1B) responses, which vary across insects. The humoral immune response begins hours after infection and involves the induction of signaling pathways (e.g., Toll, immune deficiency (IMD), and JAK–STAT signaling) in response to pattern recognition proteins or hemocyte receptors binding to pathogens. This subsequently triggers the release of antimicrobial peptides (AMPs) from the fat body and hemocytes into the hemolymph [40]. In contrast to the humoral immune response, the cellular immune response occurs rapidly following infection. Functions of the cellular immune response include melanization, pathogen encapsulation, nodulation, phagocytosis, and lysis, all of which are processes mediated by hemocytes such as granulocytes, plasmatocytes, and oenocytoids [37,40,41]. In addition to humoral and cellular immunity, insects also use RNA interference (Figure 1C) and autophagy as mechanisms to fight viral infection. 

Traditionally, the insect immune system was believed to be strictly an innate immune system with no adaptive components (e.g., antibody production as seen in mammals [42]). However, recent data have indicated the presence of “immune priming” in insects, where, following sublethal immune challenge, some insects are able to mount a strong and specific immune response (e.g., AMP production and hemocyte density) to subsequent challenge [43,44,45,46]. There is still much to be learned about immune priming in insects and associated physiological responses, and these data highlight that the insect immune system is more complex than previously believed. Of significant interest is how these different immune mediators and responses may impact the insect nervous system. Evidence exists indicating that bidirectional communication between the insect immune and nervous systems occurs (as reviewed in [47]). What currently remains unclear are the following questions: Do immune mediators play roles in regulating neuron function in insects similar to what is observed in mammals? How do systemic (e.g., hemocytes, AMPs, and corresponding signaling pathways) and local (e.g., glial cells, AMPs) immune responses impact neuron function? What are the corresponding impacts on insect behavior? To our knowledge, these questions remain relatively unanswered and underexplored; however, understanding the answers to these questions will provide invaluable insight into insect behavior and the potential relationships between infection and injury and host behavioral change. In this review, we aim to highlight some open areas of research in this field that will help in answering these questions.

## 3. Host Behavioral Change Associated with Microbial Infection

There are many different viruses, bacteria, and fungi that induce changes in the behavior of the hosts that they infect. These behavioral changes are often stereotypical, and, in many cases, the manifestation of these behavioral changes is hypothesized to aid in the survival and spread of the microbe. There is an abundance of research aimed at identifying the host- and pathogen-specific factors that, together, result in host behavioral change. However, few studies have sought to highlight the potential alterations in neuroimmune communication and glial cell signaling that may cause or contribute to changes in host behavior. Here, we discuss several examples of microbes that, following infection, cause host behavioral change, and we highlight potential avenues for future research investigating changes in host neuroimmune communication.

### 3.1. Viruses

Many viruses induce changes in behavior of the hosts that they infect. Baculoviruses [48] in particular are well-known behavioral manipulators. In species of Lepidoptera, such as *Lymantria mona*, infection with baculovirus results in hyperactivity and abnormal climbing behavior, termed “Wipfelkrankheit” or “tree-top disease” [49,50,51]. It is at the tops of trees where the larvae die and liquefy. Transmission of infectious virions can then occur via direct consumption of infected leaves or, potentially, as a consequence of rainfall [50,52]. The expression of specific baculovirus genes and alterations in phototaxis are believed to contribute to the manifestation of host behavioral change and liquification [4,5,6,7,8,53] (Table 1). At present, it is unknown whether there is also an immune component underlying changes in host behavior during baculovirus infection. Host behavioral change associated with other insect viruses has been linked to alterations in immune activation. For example, iridovirus IIV-6/cricket iridovirus (IIV-6/CrIV) infects the fat bodies of *Gryllus texensis* crickets and modifies host protein production and immune function by decreasing the expression of phenoloxidase, a virucidal enzyme [54]. Consequently, female egg production declines as well as sperm motility in infected males [54]; however, sexual behavior is maintained. IIV-6/CrIV is transmitted by physical contact; therefore, maintenance of sexual activity and courting behavior in the absence of sickness behavior, a consequence of immune activation [55,56,57,58,59,60], may be necessary for efficient spread of the virus between hosts and may be the result of decreased host production of immune proteins, immune activation, viral clearance, and sickness behavior [47]. This may also be true for *Helicoverpa zea* Nudivirus 2 (HzNv-2), a sexually transmitted virus that infects the *Helicoverpa zea* corn earworm moth. Despite gonad atrophy associated with infection [61], females infected with HzNv-2 can demonstrate enhanced and persistent calling behavior, increased contacts with males, and higher pheromone production when compared with uninfected control females [62]. The manifestation of these behaviors may help maintain viral transmission.

**Figure 2:**
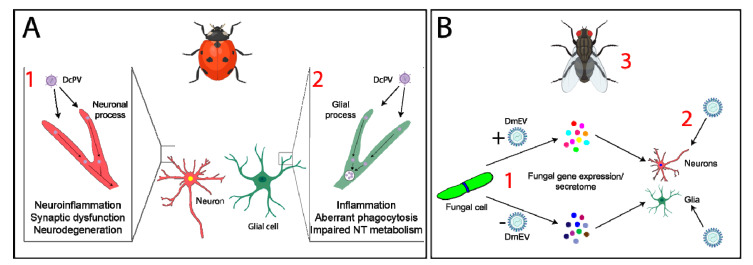
Parasites within parasites—potential impacts on neuroinflammation and glial cell function. (**A**) *Dinocampus coccinellae* paralysis virus (DcPV) is believed to trigger changes in host behavior via altering neuroinflammatory processes and glial cell function [64]. Future studies should investigate (1) if and how the virus enters neurons, mechanisms of intra-/inter-neuronal transport, effects on host neuronal gene and/or protein expression, and potential alterations in neuroinflammatory responses. These data would help provide insight into mechanisms of neuroinflammation, synaptic dysfunction, and/or neurodegeneration that may elicit changes in behavior. (2) Investigation into how glial cells respond to DcPV infection will provide data critical to understanding whether glial cells mediate neuron damage, AMP release, changes in neurotransmitter (NT) metabolism, or phagocytosis of neuronal debris in this system. (**B**) Entomophthovirus (DmEV) is associated with the entomopathogenic fungus *E. muscae*, but whether viral infection of the fungus contributes to host behavioral change remains unclear. (1) Future research should investigate whether fungal gene expression or the fungal secretome differs following DmEV infection. Additionally, whether the virus can be transferred from the fungus to host tissue (e.g., neurons or glia) causing direct effects remains to be determined (2). It is also possible that host immune responses to fungal infection may differ (3), similar to what is seen in infection with *Leishmania* parasites [111]. Images created with BioRender.com (accessed on 15 January 2021).

Given the similarities observed between the two latter systems, it is possible that decreased host anti-viral immune responses may underlie the maintenance of sexual activity in HzNv-2-infected hosts. It is also possible that viral infection may impact neuron function as a direct result of neuroinflammation, altered neuroimmune communication, and/or glial cell activation. Evidence from parasitoid systems detailing the effects of mutualistic viruses on host neuroinflammation and behavior suggests that neuroinflammation does play key roles in mediating changes in host behavior. There is an increasing number of examples of pathogens that are associated with specific parasitoids and/or other microbial pathogens (“parasites within parasites” [110]), and these smaller parasites are believed to play important roles in the success of their hosts, potentially by altering host immune reactions [64]. This has significant implications for the study of host behavioral change and the mechanisms by which it occurs, as mutualistic viruses may cause altered host immune responses, triggering changes in behavior. Recent evidence has indicated the potential importance of symbiotic viruses in initiating the stereotypical changes in behavior observed in two systems [64,68] (Figure 2). While in this review, we only discuss these two examples, an increasing number of these “parasite within parasite” relationships will likely be identified, and their existence may play an integral role in the contribution towards host behavioral change.

Some parasitoid wasps sting and alter the behavior of other insects, providing protection and/or a food source for their progeny. Proteins within the wasp’s venom may act pharmacologically in the host’s nervous system, inducing changes in host behavior [17,88,89]; however, mutualistic viruses may also play an important role. *Dinocampus coccinellae* is a parasitic wasp that lays eggs inside ladybeetles, within which the wasp larvae develop [63]. Eventually, the larvae emerge from the paralyzed ladybeetle’s body and build a cocoon between its legs. The ladybeetle serves as the larva’s bodyguard as it is rendered paralyzed. One study identified the presence of an RNA virus, named *D. coccinellae* paralysis virus (DcPV), in the heads of parasitized ladybeetles [64]. This virus, a member of the Iflaviridae family, replicates in wasp larvae and is transmitted to the ladybeetle as the larvae develop. DcPV is able to enter and replicate in the host ladybeetle nervous system and is found in glial cells in the infected ladybeetle brain, causing inflammation and/or altered glial cell function that is believed to underlie the manifestation of paralysis during the bodyguarding behavior [64]. Following viral clearance, host ladybeetles are able to recover from their paralytic state, suggesting that the virus and altered neuroinflammation and glial cell function are indeed involved in triggering host behavioral change [63,64] (Figure 2A). 

Microbe-associated viruses may also play key roles in the modulation of host behavior during infection. House flies and fruit flies infected with the entomopathogenic fungus *Entomophthora muscae* exhibit distinct changes in behavior (Table 1) [65,66,67]. Recent research has detailed the presence of a virus named *D. melanogaster* Entomophthovirus (DmEV) within *E. muscae* cells in culture and within *E. muscae* cells derived from infected *Drosophila* [68], highlighting the possibility that DmEV may play a role in regulating changes in host behavior [68]. However, further investigation is required to definitively link DmEV with host behavioral change (Figure 2B). 

Much of the current research on viruses that infect microbes indicates that these resident viruses impact host immunity following infection by the microbe. For example, viruses that infect the human parasite *Leishmania* are associated with higher incidences of treatment failure in infected humans and enhanced pathogenesis in mouse models [111,112,113,114], possibly as a consequence of altered immune control [111]. It is likely that microbe- and parasitoid-associated viruses cause alterations in host immune responses, which may contribute to changes in behavior. Compelling evidence suggests this is true of DcPV [64]. Further research is required to determine the contributions of DmEV toward host behavioral change and whether altered neuroinflammation is involved.

### 3.2. Bacteria

Unique bacterial strains within the genus *Wolbachia* number over 450 [115] and are found in approximately 70% of insects as well as in many terrestrial arthropods [116]. *Wolbachia* are vertically transmitted from an infected female to her progeny via transovarial transmission in host eggs [115,116,117,118], and infection induces reproductive changes in the host, including cytoplasmic incompatibility, parthenogenesis, feminization of genetic males, and male killing, all of which enhance its transmission [115,117,118]. *Wolbachia* is also present in the brains of infected *Drosophila* and may contribute to changes in insect behavior [119,120,121,122]. Infection with specific strains of *Wolbachia* triggers alterations in host biogenic amine synthesis, resulting in changes in activity, aggression, and sleep [123,124,125,126]. Some evidence suggests that hosts exhibit deficits in memory when infected with *Wolbachia* [127,128]. Additionally, infected hosts may exhibit changes in olfactory responsiveness [129,130] and mating behavior [131,132].

Resident bacteria populations, including the gut microbiome [133,134,135,136], play integral roles in animal behavior and communication [137,138,139,140] as well as in the maintenance of *Drosophila* homeostasis [141]. For example, gut bacteria contribute to *Drosophila* locomotor activity, and detection of bacterial cell wall components regulates egg-laying behavior, possibly by the activation of NF-κB signaling in octopaminergic neurons [142,143,144]. It is clear from these studies that host inflammatory and innate immune responses to resident bacteria play potentially important roles in modulating neuronal function and insect behavior. 

### 3.3. Fungi

There is a high diversity of fungi that are known to change host behavior [82] and, undoubtedly, more that have yet to be identified [145]. Ant pathogenic species of the *Ophiocordyceps* genus are some of the most well-studied examples of fungi that influence host behavior. Infection of *Camponotus* carpenter ants with *O. unilateralis s.l.* triggers stereotypical behavioral changes that are hypothesized to aid in fungal growth and dispersal [69,70,71,72,73,74] (Table 1). Almost identical changes in behavior are seen in *Pandora formica*-infected ants of the *Formica* genus [78,80,81,82] and *E. muscae*-infected flies [65,66,67] (as discussed above). By contrast, other entomopathogenic fungi induce distinctly different changes in host behavior. *Massospora cicadina* and *Strongwellsea castrans* infect periodical cicadas and *Hylemya brassicae* and *platura*, respectively, inducing irregular flying and crawling behavior, and in *Massospora*-infected cicadas, sexual behaviors are maintained (Table 1) [82,83,84,85].

Many studies have detailed the potential mechanisms underlying host behavioral change in these systems and have characterized changes in host gene expression, fungal production of neuroactive compounds and enterotoxins, formation of fungal networks within the host, and fungal genomes and transcriptomes [1,10,11,12,18,72,73,75,76,77,78,79,84] (see Table 1). To date, few studies have sought to specifically investigate the impacts of infection with these fungi on local host immune responses (and glial cell responses) in the brain and subsequent impacts on neuron function and host behavior. Infection with the fungus *Drechmeria coniospora* triggers increased AMP production and autophagy-mediated dendrite degeneration in *Caenorhabditis elegans* [146]. Similarly, direct inoculation of bacteria into the *Drosophila* brain causes local AMP production, neurodegeneration, and reduced locomotion [147], and infection with Zika virus induces local antiviral autophagy [148]. These data from other systems suggest that local inflammation induced by behavior-manipulating fungal infection may cause aberrant neurodegeneration and inflammatory signaling, which may impact behavior. Additionally, in *Drosophila*, bacteria located in the periphery impact behavior via modulating octopamine production, potentially by altering immune activation [125,142]. Further research, including longitudinal studies over the time course of infection, is required to determine if similar shifts in the expression of AMPs and other immune responses occur in the brains of insects infected with behavior-manipulating fungi and what impact this may have on neuron function (e.g., octopamine production). 

## 4. Parasitoids and Parasites—Neuromodulation, Molecular Mimicry, and Neuroinflammation

There are numerous examples of parasites and parasitoids that possess the ability to infect and/or manipulate the behavior of other organisms [15,149]. Parasitoids are hypothesized to utilize the synthesis and introduction of chemical neuromodulators to trigger changes in host behavior. A classic example of a behavior-manipulating parasitoid is the Jewel Wasp *Ampulex compressa*. *A. compressa* introduces venom directly into the cerebral ganglia of the *Periplaneta americana* cockroach host, leading to transient paralysis and extensive self-grooming in the cockroach [86,87]. The wasp then lays an egg on the cockroach and seals it within the burrow, where the larva can feed on the cockroach host and pupate. Direct introduction of venom into the host brain is hypothesized to cause host behavioral change via pharmacological mechanisms [16,17,89,150]. 

Similar venom-based mechanisms may be utilized by other parasitoid wasps. For example, the *Hymenoepimecis argyraphaga* wasp stings a spider host (e.g., *Plesiometa argyra*) and lays eggs on top of the spider [90,91,93]. The wasp larva then hatches, feeds on the spider, and ultimately induces the spider to build a strong cocoon web, which, following consumption of the spider, the larva uses to build a pupal cocoon. These wasps may utilize a similar method of chemical manipulation as the Jewel Wasp, given that when parasitic wasp larvae are removed from spider hosts, behavior gradually returns to normal in an apparent dose-dependent fashion [92]. 

Discussion of the relative potential impacts of shifts in a host’s neuroinflammatory state in response to interactions with a parasitoid (with the exception of *D. coccinellae*, as discussed above) remains relatively limited. As the *A. compressa* wasp stings the cockroach directly in the target ganglia [86], there is likely a corresponding inflammatory response (and perhaps glial cell response) that occurs in response to injury. This hypothesis is supported by observations from *Drosophila* models of stab injury and traumatic brain injury. Following stab injury, glial cell division increases in the brains of *D. melanogaster* [151]. Additionally, in *Drosophila* subjected to traumatic brain injury, the induction of innate immune signaling pathways (e.g., Toll receptor and IMD signaling pathways), disruption of the blood–brain barrier, and increased expression of AMPs (e.g., *metchnikowin*, *attacin C*, *diptericin B*, and *spz*) is evident [29,152,153,154]. Similar alterations in innate immune activation, AMP expression, blood–brain barrier disruption, and glial cell division and/or activation may also contribute to altered host behavior and locomotion in parasitoid systems, possibly as a consequence of neurodegeneration and neurotoxicity, as has been observed in *Drosophila* models (e.g., Alzheimer’s disease, aging, amyotrophic lateral sclerosis, and polyglutamine disease, among others) [29,30]. Further investigation into these processes may reveal whether inflammation as a result of a parasitoid sting contributes to overt neuron degeneration and/or altered neuronal communication due to aberrant synaptic activity or pruning, which may contribute to altered neuron function and subsequent behavioral change. 

It is possible that inflammatory responses may also occur in the *P. argyra* spider system. This hypothesis is supported by the fact that affected spiders can recover when wasp larvae are removed [92]. While manifestation of host behavioral change occurred within a few days, behavioral recovery took up to 2 weeks. This finding could, as the authors hypothesized, indicate slow removal and/or degradation of a larva-derived substance that directly impacts the host nervous system and behavior. Alternatively (or concordantly), association of the parasitoid wasp larva with the spider host may trigger an inflammatory response that impacts nervous tissue. Any neuroinflammation induced by the parasitoid larva would take time to recover from, similar to what is observed during DcPV infection [64,155]. Taken together, it remains unknown if neuroinflammation is a contributing factor towards host behavioral change in parasitoid–host systems such as those described here. Studies investigating the cellular and molecular effects of these associations are limited. Future studies could investigate whether neuroinflammation occurs in these systems and whether the induction of such processes contributes to host behavioral change. 

Similar to parasitoids, some parasites may utilize chemical mechanisms and neuromodulation to impact the behavior of their hosts. Juvenile hairworms such as *Spinochordodes tellinii* and *Paragordius tricuspidatus* infect grasshoppers and crickets, respectively, and infected hosts are often observed jumping into water [94]. Hairworms of both grasshoppers (*S. tellinii*) and crickets (*P. tricuspidatus*) produce mimetic molecules similar to proteins involved in Wnt signaling and secrete proteins associated with neurotransmission and the regulation of apoptosis [13,14]. Additionally, the parasitic worms *Euhaplorchis californiensis* and *Microphallus papillorobustus* infect and alter the behavior of killifish and gammarids, respectively, causing changes in behavior that increase the probability of consumption by terminal hosts (Table 1). These latter changes in behavior are hypothesized to be triggered by changes in monoamine signaling in the host [19,20,95,96,97,98,99,100].

Similar to parasitoid–host systems, the contributions of neuroinflammation towards host behavioral changes in response to infection with the parasites mentioned here are relatively unclear and understudied. Changes in neuromodulation triggered by *M. papillorobustus* infection may be due, in part, to an altered neuroinflammatory state and glial cell activation in the host [100]. Specifically, a subset of parasite metacercariae demonstrated variable levels of melanization, and, at the host–parasite interface, nitric oxide synthase (NOS) levels are elevated and astrocyte-like glial cells and their processes are present [100]. These shifts in neuroinflammation and glial cell activation may directly impact host behavior or may indirectly alter host behavior by impacting neuronal monoamine synthesis and signaling. In support of this hypothesis, the well-known *Toxoplasma gondii*–rodent system possesses a neuroinflammatory component to host behavioral change that may be more critical than other well-documented mechanisms [3,101,102,103,104,105,106,107,108,109]. 

While *E. californiensis*, *M. papillorobustus*, and *T. gondii* do not infect insects, they provide important context for the interpretation of the molecular mechanisms underlying host behavioral change in insects. It is clear that in the former systems, neuroinflammation may play key roles in mediating shifts in host behavior. It is very likely that this is also true in insect systems and may be mediated by similar mechanisms (e.g., glial cell activation or altered immune mediator synthesis). Targeted research in insects is required to determine if aberrant immune signaling and/or neuroinflammation occurs in the nervous systems of affected hosts and to link any alterations in immune signaling with changes in host behavior.

## 5. Neuroinflammation and Glia

In mammals, there is a clear link between the expression of different immune mediators and changes in neuron function, signaling, and development (e.g., cytokines/chemokines, major histocompatibility complex signaling, and complement signaling, etc.) [22,24,25,26,27]. Recent data from *Drosophila* models of neurodegenerative disease, nervous system infection, and traumatic brain injury have indicated that similar processes may contribute to neuron health in insects [29,30,143]. For example, local alterations in Toll and IMD pathway signaling and the expression of specific AMP genes (e.g., *drosomycin*, *defensin*, and *metchnikowin*) are hypothesized to be involved in neurodegeneration and neurotoxicity [29,30,152,153,156,157,158]. Additionally, insect inflammatory mediators, including NF-κB/IMD pathway activation and AMP production, contribute to the maintenance of normal neuron functions in insects and regulate neurotransmitter release at the neuromuscular junction [159,160], sleep [161,162,163,164], and memory formation [143,165].

**Figure 3:**
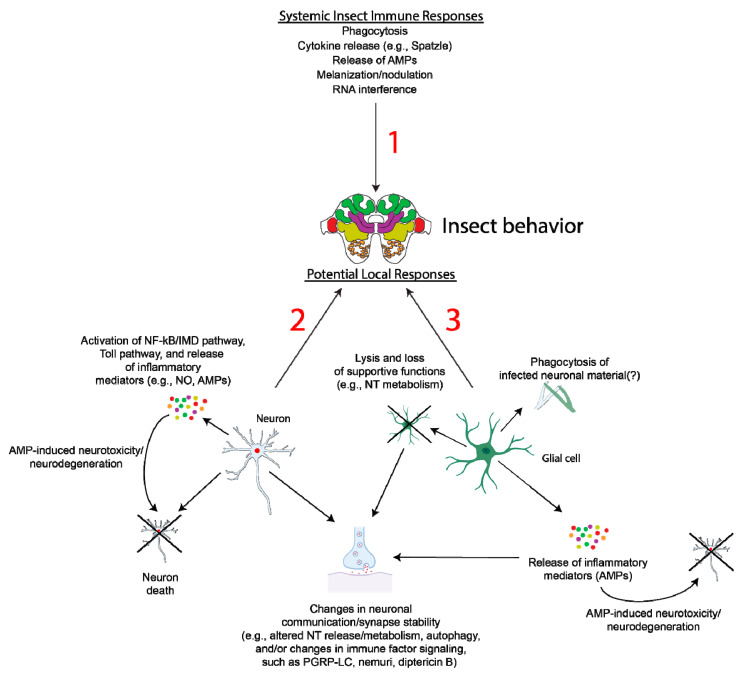
Potential alterations in local immune responses and relationship with insect behavior. While there is the potential for cross-talk between immune modulators such as AMPs, cytokines, etc., and the nervous system (1), the detailed existence of this cross-talk as well as any impacts on insect behavior in behavioral manipulator insect systems remain to be fully detailed. During infection by a behavioral manipulator (e.g., *O. kimflemingiae*) or interaction with a parasitoid (e.g., *A. compressa*), local inflammatory reactions by neurons (2) and/or glia (3) to pathogens or parasitoid-related injury may cause neuron damage and degeneration, potentially via the release of AMPs (e.g., metchnikown, drosomysin, and defensin). Degeneration of glia may cause loss of supportive functions and altered neurotransmitter (NT) release or metabolism. Additionally, specific AMPs are functionally pleiotropic in *Drosophila*, including peptidoglycan recognition protein LC (PGRP-LC), nemuri, and diptericin B, which contribute to the regulation of presynaptic homeostatic plasticity, sleep, and memory formation, respectively [159,163,165]. Alterations in the expression of such AMPs by neurons and/or glia may, therefore, directly impact neuron communication and the regulation of synaptic stability. Changes in neuronal autophagy, as has been observed in Zika-infected *Drosophila* [148], may also impact synapse structure, as autophagy plays key roles in synapse development [173]. It is unclear whether infection or injury triggers aberrant glia-dependent phagocytosis of neuronal material and this requires further investigation. These hypotheses remain to be tested in the models described here and may prove to be exciting areas for future research in the field of behavioral manipulation in insects. Images created with BioRender.com (accessed on 15 January 2021).

Together, these data suggest that similar inflammation-based processes may regulate homeostatic neuronal processes as well as neurodegeneration and neurotoxicity in insect systems, similar to what is observed in mammals. It is, therefore, possible that altered neuroinflammation may indeed be a contributing factor toward the behavioral changes observed in a multitude of different parasite/parasitoid–host systems. However, little is known about the potential inductions in neuroinflammation and/or shifts in glial cell function in insects affected by behavioral manipulators (Figure 3). Free radical production, such as the production of nitric oxide (NO), during infection may contribute to host behavioral change. NO is an immune effector molecule in both vertebrates and invertebrates that is generated via the activation of NOS [166]. NOS is expressed in neurons [167], and, as discussed above, in *M. papillorobustus*-infected gammarids, NOS immunoreactivity is increased at the parasite–host interface [100]. NO plays alternative roles in neurotransmission and neuromodulation in both mammals and invertebrates [167]; therefore, altered synthesis of NO triggered as an immune response may contribute to altered neurotransmission and/or neuron function and, ultimately, host behavior. Other immune mediators may also play roles. For example, the circulating cytokine Spätzle binds to Toll receptors and contributes to innate immune defense processes in *Drosophila* [168]. Additional evidence demonstrates that Spätzle is also a member of neurotrophin-like signaling molecules and functions as a neurotrophin during invertebrate development and in synaptogenesis [169,170,171]. It is possible that if a cytokine such as Spätzle were to change in expression in response to infection, there may be an impact on synaptic function. However, this hypothesis remains to be tested.

Some aspects of insect immune activation may not cause changes in behavior, or may cause changes in behavior only in a subset of cases. For example, melanization of cerebral-localized *M. papillorobustus* metacercariae effectively stops the initiation of any host behavioral changes triggered by *M. papillorobustus* infection in gammarids [100,172]. Melanization may, therefore, be an effective immune strategy to fight infection and stop host behavioral change, and behavioral change may instead manifest as the result of parasite-specific factors and/or alternative host immune responses, including the release of AMPs. However, this may not necessarily be true in other systems, including in insects, and further research is required to determine what cells in the nervous system and what mechanisms underlie melanization in the infected brain, as, to date, this is still unclear in the gammarid system [100].

It is of note that in the *O. unilateralis s.l.* system, behavioral change occurs in a circadian-controlled manner. Data indicate that, in accordance with the circadian-controlled manifestation of host behavioral change, the *O. kimflemingiae* transcriptome also exhibits circadian-controlled shifts in gene expression, which likely play a role in modulating host behavior [12]. Many immune-related genes are under circadian control in *Drosophila* [174], and time of day of bacterial infection in *Drosophila* affects bacterial load and host immune competence [175]. We can hypothesize that the expression of immune mediators in, for example, *O. kimflemingiae*-infected ants may also be under circadian control and may play roles in triggering host climbing and biting. This is supported by evidence demonstrating the rhythmic expression of the gene *Achilles* in the *Drosophila* brain, which represses the expression of AMPs, including *metchnikown* and *drosomysin* [176]. Local rhythmic expression of AMPs combined with the expression of fungal-derived factors may, together, produce the stereotypical changes in behavior observed in the *O. unilateralis* system. However, this is only a hypothesis, and future studies should investigate whether circadian-controlled modulation of AMP expression also occurs in other insects, including *C. castaneus* ants, and how this may impact behavior. 

Alterations in glial cell signaling and inductions in glial cell-based inflammatory responses during infection may play key roles in mediating changes in neuron function and behavior during infection in insects. There are six major types of glial cells in the insect nervous system, including astrocytes, cell body glia, sub-perineurial glia, perineurial glia, wrapping glia, and ensheathing glia [31]. These cells can be categorized based on morphology and localization as surface glia, cortex glia, and neuropil glia [31,177,178]. Much of the existing data on insect glial cell function come from *Drosophila* models [33,34,177,178,179,180,181]. We can gather, from these studies, that glial cells in insects function in much the same way as glial cells in mammals, including providing neurons with metabolic support critical for neurotransmission [31], participating in synapse remodeling [32], and clearing neuronal debris via phagocytosis [33,34,35]. What currently remains unclear is whether insect glia play roles during interactions with behavioral manipulators, and, if they do, what glial cell-based mechanisms mediate pathogen clearance/injury response and whether these processes impact neuron function and behavior.

Data from *Drosophila* models demonstrate increased expression of inflammatory mediators in the heads of flies subjected to traumatic brain injury and in fly models of various neurodegenerative diseases [29,30]. Additionally, direct inoculation of the *Drosophila* brain with bacteria triggers increased gene expression of the AMP *attacin* and infection with Zika virus causes activation of the IMD pathway, increased *diptericin* expression, and autophagy in infected fly heads [29,147,148]. Together, these data demonstrate that the insect nervous system is capable of mounting a local innate immune response to pathogens and that induction of these pathways may lead to neurodegeneration, aberrant neuron function, and altered behavior. However, it remains unclear whether the increased expression of AMPs and innate immune signaling pathways observed in many of these models may be from neurons, glial cells, or both. The expression of AMPs in glial cells is evident in a fly model of ataxia-telangiectasia, and dysregulation of the glial inflammatory response may mediate neurodegeneration [29,182]. This is also true in aged flies, where overactivation of NF-κB/IMD pathway-dependent AMP production in glial cells causes neurodegeneration, deficits in locomotion, and decreased lifespan [143,183]. Evidence from DcPV-infected ladybeetles and parasite-infected invertebrates suggests inflammatory responses and glial cell activation may be involved in host behavioral change [64,100]. It is, therefore, likely that altered glial cell activation, Toll and/or IMD pathway signaling, and AMP expression may lead to neurodegeneration and alterations in behavior in behavioral manipulator systems. Of interest is whether glial cells contribute to phagocytosis of infected material in the host brain (Figure 3), similar to what occurs when glial cells phagocytose neuronal debris [33,34,35]. This may lead to aberrant synaptic pruning and phagocytosis of neuronal material, which may alter neuronal signaling and lead to host behavioral change. 

Overall, there is a paucity of data regarding the activity of insect glial cells in behavioral manipulator models. The parasitoid wasp-derived virus DcPV localizes in the cytoplasm of glia in the infected ladybeetle brain, which may impact neuron function and/or neuroinflammation as a result of glial cell lysis [64]. Additionally, glial cells may play roles in mediating neuron recovery in this model [64]. Data from *Drosophila* show that local innate immune responses occur in the brain in response to infection [147], which may be mediated by glial cells that are known to express AMPs resulting in neurodegeneration [182]. Given these data and the parallels in glial cell function between insects and mammals (for example, serving phagocytic and supportive metabolic functions that may impact neuron signaling and function [31,34]), we could hypothesize that during infection, there may be an induction of glial cell activation and/or aberrant metabolic activity that may cause dysregulation of insect neurotransmitter metabolism and/or and the release of inflammatory factors (Figure 3). These processes may collectively result in neuron damage, altered neuron communication, neuroinflammation, and potentially altered behavior in behavioral manipulator models. However, data definitively linking neuroinflammatory processes and glial cell activation to changes in neurotransmission, neuron function, and behavioral change in this model are lacking. It is also possible that changes in local inflammation (e.g., NO release [100]) may trigger aberrant phagocytic activity by glial cells [33,34]. Alterations in neuronal autophagy may also cause changes in synapse dynamics [148,173]. It is clear from *Drosophila* models that inflammatory mediators and immune pathways, including AMPs and NF-κB/IMD pathway activity, can possess functional pleiotropy in insects, as they contribute to the maintenance of normal host behaviors (e.g., neuromuscular junction neurotransmission, sleep, and memory) [143,159,160,161,162,163,164,165]. It is, therefore, possible that altered expression of inflammatory factors in insects during infection by a behavioral manipulator may directly contribute to altered neurotransmission or synaptic function (Figure 3). Further investigation into the mechanisms underlying how immune factors (e.g., AMPs) may more subtly impact neuron function (versus causing overt neurodegeneration/neurotoxicity) is required. 

While we can hypothesize, based on current data in *Drosophila* and behavior-manipulating models, that glial cell function and neuroinflammation may become dysregulated in the behavioral manipulator models discussed here, leading to aberrant neuron function, this is purely speculative and needs to be thoroughly tested in the lab. It is clear that glial cells are affected during infection in models of behavioral manipulation [64,100]. It is likely that glial cells play important and integral roles during infection by a behavior-manipulating microbe and/or injury by a parasitoid, potentially by impacting neuron function through altered metabolism, neurotransmitter synthesis, and clearance and/or through neuroinflammation, which may directly impact neuron function and signaling or induce phagocytosis. More research is clearly needed in this area. Future research investigating the effects of infection on glial cell function in insects will provide invaluable information regarding neuron–glia communication during immune challenge and the contributions of glial cells toward neuron function and, ultimately, the manifestation of behavior in insects.

## 6. Conclusions

A common theme amongst all of the examples discussed here is that there is still much we do not know about the mechanisms underlying how different organisms influence host behavior. Many of these systems remain poorly understudied. Altered inflammatory responses and neuroimmune communication are common consequences of infection and injury, and their contributions towards host behavioral change cannot be discounted. The goal of this review was not to say that neuroinflammation is the sole cause of altered behavior in the examples discussed but rather to highlight the fact that neuroinflammation and immune reactions that occur as consequences of infection and/or parasitoid interactions are factors that cannot be ignored and may, in fact, play key roles in mediating host behavioral change, particularly at the glia–neuron interface. 

The data discussed here illustrate that there is likely no single mechanistic answer underlying how an organism is able to influence the behavior of its host, and more than one mechanism is likely employed (e.g., the introduction of neuromodulators combined with altered neuroimmune communication or glial cell activation, etc.). Studying the mechanisms underlying host behavioral change can yield invaluable insight on microbe–host co-evolution, mechanisms of microbial pathogenesis, neurological disease, animal behavior, and infectious disease spread within a population [184,185,186,187,188,189]. To date, few studies have sought to detail the potential impacts of altered host immune responses, neuroimmune communication, and glial cell responses on host behavior in model systems such as those discussed here. Behavioral manipulators may provide excellent platforms in which to study insect neuroimmune communication and the impacts of neuroinflammation on insect behavior. By conducting studies such as those proposed here, we will deepen our understanding of insect neurobiology, better understand the potential effects of insect neuroinflammation on behavior, and elucidate the potential roles of insect glial cells on mediating these processes.

## Figures and Tables

**Table 1:** Host–parasite/parasitoid systems, behavioral changes, and hypothesized mechanisms^1^.

Host	Parasite	Behavior	Mechanism(s)	Refs
Lepidoptera, Diptera, Hymenoptera, etc.	Baculoviruses	Tree climbing, enhanced locomotor activity and liquification	Expression of virus-specific genes, *egt*, *ptp*, chitinase, and cathepsin	[4,5,6,7,8,48,49,50,51,52,53]
*Gryllus texensis*	IIV-6/CrIV	Decreased egg production and sperm motility; maintained sexual behavior	Decreased expression of the virucidal enzyme phenoloxidase	[54]
*Helicoverpa zea*	HzNv-2	Persistent calling behavior; increased pheromone production and contacts with males in infected females	Unknown	[61,62]
Ladybeetle	*Dinocampus coccinellae*/ DcPV	Bodyguard behavior/paralysis	Viral replication in host nervous tissue; neuroinflammation	[63,64]
House flies/fruit flies	*Entomophthora muscae*/DmEV	Attach to an elevated surface via the proboscides and raise wings, allowing for fungal spore dispersal	Unknown	[65,66,67,68]
Ants (*Camponotus*)	*Ophiocordyceps unilateralis*	Impaired motor control, convulsions; travel to areas optimal for fungal growth; circadian-synchronized biting behavior	Fungal production of enterotoxins and neuroactive compounds; altered host gene expression; formation of dense fungal network	[1,11,12,18,69,70,71,72,73,74,75,76,77]
Ants (*Formica*)	*Pandora formica*	Summit disease and biting behavior	Unknown	[78,79,80,81,82]
Periodical cicadas	*Massospora cicadina* *Massospora platypediae* *Massospora levispora*	Irregular flying and crawling behavior; altered sexual behavior	*M. cicadina*—production of the plant amphetamine cathinone; *M. platypediae* and *M. levispora*—production of psilocybin	[10,82,83,84]
*Hylemya brassicae Hylemya platura*	*Strongwellsea castrans*	Irregular flying and crawling behavior	Unknown	[85]
*Periplaneta americana*	*Ampulex compressa*	Transient paralysis and self-grooming	Venom containing neuromodulators	[16,17,86,87,88,89]
*Plesiometa argyra*	*Hymenoepimecis argyraphaga*	Altered web construction	Hypothesized venom-based modulation	[90,91,92,93]
Grasshoppers/crickets	*Spinochordodes tellinii/* *Paragordius tricuspidatus*	Water-seeking behavior	Production of mimetic molecules (e.g., Wnt signaling modulators) and proteins involved in neurotransmission and apoptosis	[13,14,94]
Killifish	*Euhaplorchis californiensis*	Rapid swimming and “flashing”, increasing predation by birds	Altered monoamine signaling	[19,20,95,96]
Gammarids	*Microphallus papillorobustus*	Altered responses to environmental stimuli, increasing predation by birds	Altered monoamine signaling and neuroinflammation (i.e., NO synthesis)	[97,98,99,100]
Rodents	*Toxoplasma gondii*	Altered innate fear responses to cat odor	Alterations in dopamine and testosterone synthesis, epigenetic modifications, cyst formation, and neuroinflammation	[3,101,102,103,104,105,106,107,108,109]

Abbreviations: DcPV, *Dinocampus coccinellae* paralysis virus; DmEV, *Drosophila melanogaster* Entomophthovirus; HzNv-2, *Helicoverpa zea* Nudivirus 2; IIV-6/CrIV, iridovirus IIV-6/cricket iridovirus; NO, nitric oxide.

## Data Availability

Not applicable

## References

[1] de Bekker C., Ohm R.A., Evans H.C., Brachmann A., Hughes D.P. (2017). Ant-Infecting Ophiocordyceps Genomes Reveal a High Diversity of Potential Behavioral Manipulation Genes and a Possible Major Role for Enterotoxins. Sci. Rep..

[2] Kobmoo N., Wichadakul D., Arnamnart N., Vega R.C.R.D.L., Luangsa-Ard J.-J., Giraud T. (2018). A Genome Scan of Diversifying Selection in Ophiocordyceps Zombie-Ant Fungi Suggests a Role for Enterotoxins in Coevolution and Host Specificity. Mol. Ecol..

[3] Gaskell E.A., Smith J.E., Pinney J.W., Westhead D.R., McConkey G.A. (2009). A Unique Dual Activity Amino Acid Hydroxylase in Toxoplasma Gondii. PLoS ONE.

[4] Kamita S.G., Nagasaka K., Chua J.W., Shimada T., Mita K., Kobayashi M., Maeda S., Hammock B.D. (2005). A Baculovirus-Encoded Protein Tyrosine Phosphatase Gene Induces Enhanced Locomotory Activity in a Lepidopteran Host. Proc. Natl. Acad. Sci. USA.

[5] Katsuma S., Koyano Y., Kang W., Kokusho R., Kamita S.G., Shimada T. (2012). The Baculovirus Uses a Captured Host Phosphatase to Induce Enhanced Locomotory Activity in Host Caterpillars. PLoS Pathog..

[6] Hoover K., Grove M., Gardner M., Hughes D.P., McNeil J., Slavicek J. (2011). A Gene for an Extended Phenotype. Science.

[7] Han Y., van Houte S., Drees G.F., van Oers M.M., Ros V.I.D. (2015). Parasitic Manipulation of Host Behaviour: Baculovirus SeMNPV EGT Facilitates Tree-Top Disease in Spodoptera Exigua Larvae by Extending the Time to Death. Insects.

[8] Hawtin R.E., Zarkowska T., Arnold K., Thomas C.J., Gooday G.W., King L.A., Kuzio J.A., Possee R.D. (1997). Liquefaction of Autographa Californica Nucleopolyhedrovirus-Infected Insects Is Dependent on the Integrity of Virus-Encoded Chitinase and Cathepsin Genes. Virology.

[9] Adamo S.A. (2013). Parasites: Evolution’s Neurobiologists. J. Exp. Biol..

[10] Boyce G.R., Gluck-Thaler E., Slot J.C., Stajich J.E., Davis W.J., James T.Y., Cooley J.R., Panaccione D.G., Eilenberg J., De Fine Licht H.H. (2019). Psychoactive Plant- and Mushroom-Associated Alkaloids from Two Behavior Modifying Cicada Pathogens. Fungal Ecol..

[11] de Bekker C., Ohm R.A., Loreto R.G., Sebastian A., Albert I., Merrow M., Brachmann A., Hughes D.P. (2015). Gene Expression during Zombie Ant Biting Behavior Reflects the Complexity Underlying Fungal Parasitic Behavioral Manipulation. BMC Genom..

[12] de Bekker C., Will I., Hughes D.P., Brachmann A., Merrow M. (2017). Daily Rhythms and Enrichment Patterns in the Transcriptome of the Behavior-Manipulating Parasite Ophiocordyceps Kimflemingiae. PLoS ONE.

[13] Biron D.G., Ponton F., Marché L., Galeotti N., Renault L., Demey-Thomas E., Poncet J., Brown S.P., Jouin P., Thomas F. (2006). ‘Suicide’ of Crickets Harbouring Hairworms: A Proteomics Investigation. Insect Mol. Biol..

[14] Biron D.G., Marché L., Ponton F., Loxdale H.D., Galéotti N., Renault L., Joly C., Thomas F. (2005). Behavioural Manipulation in a Grasshopper Harbouring Hairworm: A Proteomics Approach. Proc. Biol. Sci..

[15] Hughes D.P., Brodeur J., Thomas F. (2012). Host Manipulation by Parasites.

[16] Kaiser M., Arvidson R., Zarivach R., Adams M.E., Libersat F. (2019). Molecular Cross-Talk in a Unique Parasitoid Manipulation Strategy. Insect Biochem. Mol. Biol..

[17] Kaiser M., Libersat F. (2015). The Role of the Cerebral Ganglia in the Venom-Induced Behavioral Manipulation of Cockroaches Stung by the Parasitoid Jewel Wasp. J. Exp. Biol..

[18] Loreto R.G., Hughes D.P. (2019). The Metabolic Alteration and Apparent Preservation of the Zombie Ant Brain. J. Insect Physiol..

[19] Shaw J.C., Korzan W.J., Carpenter R.E., Kuris A.M., Lafferty K.D., Summers C.H., Øverli Ø. (2009). Parasite Manipulation of Brain Monoamines in California Killifish (*Fundulus parvipinnis*) by the Trematode Euhaplorchis Californiensis. Proc. Biol. Sci..

[20] Shaw J.C., Øverli Ø. (2012). Brain-Encysting Trematodes and Altered Monoamine Activity in Naturally Infected Killifish *Fundulus parvipinnis*. J. Fish Biol..

[21] Morimoto K., Nakajima K. (2019). Role of the Immune System in the Development of the Central Nervous System. Front. Neurosci..

[22] Hong S., Dissing-Olesen L., Stevens B. (2016). New Insights on the Role of Microglia in Synaptic Pruning in Health and Disease. Curr. Opin. Neurobiol..

[23] Mottahedin A., Ardalan M., Chumak T., Riebe I., Ek J., Mallard C. (2017). Effect of Neuroinflammation on Synaptic Organization and Function in the Developing Brain: Implications for Neurodevelopmental and Neurodegenerative Disorders. Front. Cell Neurosci..

[24] Boulanger L.M. (2009). Immune Proteins in Brain Development and Synaptic Plasticity. Neuron.

[25] Shatz C.J. (2009). MHC Class I: An Unexpected Role in Neuronal Plasticity. Neuron.

[26] Stephan A.H., Barres B.A., Stevens B. (2012). The Complement System: An Unexpected Role in Synaptic Pruning during Development and Disease. Annu. Rev. Neurosci..

[27] Yirmiya R., Goshen I. (2011). Immune Modulation of Learning, Memory, Neural Plasticity and Neurogenesis. Brain Behav. Immun..

[28] Lucin K.M., Wyss-Coray T. (2009). Immune Activation in Brain Aging and Neurodegeneration: Too Much or Too Little?. Neuron.

[29] Lye S.H., Chtarbanova S. (2018). Drosophila as a Model to Study Brain Innate Immunity in Health and Disease. Int. J. Mol. Sci..

[30] Nainu F., Salim E., Asri R.M., Hori A., Kuraishi T. (2019). Neurodegenerative Disorders and Sterile Inflammation: Lessons from a Drosophila Model. J. Biochem..

[31] Rittschof C.C., Schirmeier S. (2018). Insect Models of Central Nervous System Energy Metabolism and Its Links to Behavior. Glia.

[32] Fuentes-Medel Y., Logan M.A., Ashley J., Ataman B., Budnik V., Freeman M.R. (2009). Glia and Muscle Sculpt Neuromuscular Arbors by Engulfing Destabilized Synaptic Boutons and Shed Presynaptic Debris. PLoS Biol..

[33] Freeman M.R. (2015). Drosophila Central Nervous System Glia. Cold Spring Harb. Perspect. Biol..

[34] Kurant E. (2011). Keeping the CNS Clear: Glial Phagocytic Functions in Drosophila. Glia.

[35] Purice M.D., Ray A., Münzel E.J., Pope B.J., Park D.J., Speese S.D., Logan M.A. (2017). A Novel Drosophila Injury Model Reveals Severed Axons Are Cleared through a Draper/MMP-1 Signaling Cascade. eLife.

[36] Dushay M. (2010). Insect Infection and Immunity. Evolution, Ecology, and Mechanisms. Jens Rolff and Stuart Reynolds, Editors. Integr. Comp. Biol..

[37] Hillyer J.F. (2016). Insect Immunology and Hematopoiesis. Dev. Comp. Immunol..

[38] Siva-Jothy M.T., Moret Y., Rolff J., Simpson S.J. (2005). Insect Immunity: An Evolutionary Ecology Perspective. Advances in Insect Physiology.

[39] Cremer S., Armitage S.A.O., Schmid-Hempel P. (2007). Social Immunity. Curr. Biol..

[40] Rosales C. (2017). Cellular and Molecular Mechanisms of Insect Immunity. Insect Physiol. Ecol..

[41] Lawrence P.O., Capinera J.L. (2008). Hemocytes of Insects: Their Morphology and Function. Encyclopedia of Entomology.

[42] Flajnik M.F., Kasahara M. (2010). Origin and Evolution of the Adaptive Immune System: Genetic Events and Selective Pressures. Nat. Rev. Genet..

[43] Cooper D., Eleftherianos I. (2017). Memory and Specificity in the Insect Immune System: Current Perspectives and Future Challenges. Front. Immunol..

[44] Ferro K., Peuß R., Yang W., Rosenstiel P., Schulenburg H., Kurtz J. (2019). Experimental Evolution of Immunological Specificity. Proc. Natl. Acad. Sci. USA.

[45] Sadd B.M., Schmid-Hempel P. (2006). Insect Immunity Shows Specificity in Protection upon Secondary Pathogen Exposure. Curr. Biol..

[46] Sheehan G., Farrell G., Kavanagh K. (2020). Immune Priming: The Secret Weapon of the Insect World. Virulence.

[47] Adamo S.A., Beckage N.E. (2008). Bidirectional connections between the immune system and the nervous system in insects. Insect Immunology.

[48] Herniou E.A., Olszewski J.A., Cory J.S., O’Reilly D.R. (2003). The Genome Sequence and Evolution of Baculoviruses. Annu. Rev. Entomol..

[49] Hofmann R. (1891). Insektentötende Pilze mit Besonderer Berücksichtigung der “Nonne”.

[50] Goulson D. (1997). Wipfelkrankheit: Modification of Host Behaviour during Baculoviral Infection. Oecologia.

[51] Heil M. (2016). Host Manipulation by Parasites: Cases, Patterns, and Remaining Doubts. Front. Ecol. Evol..

[52] D’Amico V., Elkinton J.S. (1995). Rainfall Effects on Transmission of Gypsy Moth (Lepidoptera: Lymantriidae) Nuclear Polyhedrosis Virus. Environ. Entomol..

[53] van Houte S., van Oers M.M., Han Y., Vlak J.M., Ros V.I.D. (2014). Baculovirus Infection Triggers a Positive Phototactic Response in Caterpillars to Induce ‘Tree-Top’ Disease. Biol. Lett..

[54] Adamo S.A., Kovalko I., Easy R.H., Stoltz D. (2014). A Viral Aphrodisiac in the Cricket Gryllus Texensis. J. Exp. Biol..

[55] Adamo S.A. (2012). The Effects of the Stress Response on Immune Function in Invertebrates: An Evolutionary Perspective on an Ancient Connection. Horm. Behav..

[56] Aubert A. (1999). Sickness and Behaviour in Animals: A Motivational Perspective. Neurosci. Biobehav. Rev..

[57] Dantzer R. (2004). Cytokine-Induced Sickness Behaviour: A Neuroimmune Response to Activation of Innate Immunity. Eur. J. Pharm..

[58] Dantzer R., Kelley K.W. (1989). Stress and Immunity: An Integrated View of Relationships between the Brain and the Immune System. Life Sci..

[59] Hart B.L. (1988). Biological Basis of the Behavior of Sick Animals. Neurosci. Biobehav. Rev..

[60] Sullivan K., Fairn E., Adamo S.A. (2016). Sickness Behaviour in the Cricket Gryllus Texensis: Comparison with Animals across Phyla. Behav. Process..

[61] Raina A.K., Adams J.R. (1995). Gonad-Specific Virus of Corn Earworm. Nature.

[62] Burand J.P., Tan W., Kim W., Nojima S., Roelofs W. (2005). Infection with the Insect Virus Hz-2v Alters Mating Behavior and Pheromone Production in Female Helicoverpa Zea Moths. J. Insect Sci..

[63] Maure F., Brodeur J., Ponlet N., Doyon J., Firlej A., Elguero É., Thomas F. (2011). The Cost of a Bodyguard. Biol. Lett..

[64] Dheilly N.M., Maure F., Ravallec M., Galinier R., Doyon J., Duval D., Leger L., Volkoff A.-N., Missé D., Nidelet S. (2015). Who Is the Puppet Master? Replication of a Parasitic Wasp-Associated Virus Correlates with Host Behaviour Manipulation. Proc. R. Soc. Lond. B Biol. Sci..

[65] Krasnoff S.B., Watson D.W., Gibson D.M., Kwan E.C. (1995). Behavioral Effects of the Entomopathogenic Fungus, Entomophthora Muscae on Its Host Musca Domestica: Postural Changes in Dying Hosts and Gated Pattern of Mortality. J. Insect Physiol..

[66] Goldstein B. (1927). An Empusa Disease of Drosophila. Mycologia.

[67] Elya C., Lok T.C., Spencer Q.E., McCausland H., Martinez C.C., Eisen M. (2018). Robust Manipulation of the Behavior of Drosophila Melanogaster by a Fungal Pathogen in the Laboratory. eLife.

[68] Coyle M.C., Elya C.N., Bronski M.J., Eisen M.B. (2018). Entomophthovirus: An Insect-Derived Iflavirus That Infects a Behavior Manipulating Fungal Pathogen of Dipterans. bioRxiv.

[69] Araújo J.P.M., Hughes D.P. (2017). The Fungal Spore: Its Organization and Role in the Ecosystem. The Fungal Community: Its Organization and Role in the Ecosystem.

[70] Andersen S.B., Gerritsma S., Yusah K.M., Mayntz D., Hywel-Jones N.L., Billen J., Boomsma J.J., Hughes D.P. (2009). The Life of a Dead Ant: The Expression of an Adaptive Extended Phenotype. Am. Nat..

[71] de Bekker C. (2019). Ophiocordyceps–Ant Interactions as an Integrative Model to Understand the Molecular Basis of Parasitic Behavioral Manipulation. Curr. Opin. Insect Sci..

[72] de Bekker C., Quevillon L.E., Smith P.B., Fleming K.R., Ghosh D., Patterson A.D., Hughes D.P. (2014). Species-Specific Ant Brain Manipulation by a Specialized Fungal Parasite. BMC Evol. Biol..

[73] Hughes D.P., Andersen S.B., Hywel-Jones N.L., Himaman W., Billen J., Boomsma J.J. (2011). Behavioral Mechanisms and Morphological Symptoms of Zombie Ants Dying from Fungal Infection. BMC Ecol..

[74] Loreto R.G., Araújo J.P.M., Kepler R.M., Fleming K.R., Moreau C.S., Hughes D.P. (2018). Evidence for Convergent Evolution of Host Parasitic Manipulation in Response to Environmental Conditions. Evolution.

[75] Fredericksen M.A., Zhang Y., Hazen M.L., Loreto R.G., Mangold C.A., Chen D.Z., Hughes D.P. (2017). Three-Dimensional Visualization and a Deep-Learning Model Reveal Complex Fungal Parasite Networks in Behaviorally Manipulated Ants. Proc. Natl. Acad. Sci. USA.

[76] Mangold C.A., Ishler M.J., Loreto R.G., Hazen M.L., Hughes D.P. (2019). Zombie Ant Death Grip Due to Hypercontracted Mandibular Muscles. J. Exp. Biol..

[77] Will I., Das B., Trinh T., Brachmann A., Ohm R.A., de Bekker C. (2020). Genetic Underpinnings of Host Manipulation by Ophiocordyceps as Revealed by Comparative Transcriptomics. G3 Genes Genomes Genet.

[78] Małagocka J., Grell M.N., Lange L., Eilenberg J., Jensen A.B. (2015). Transcriptome of an Entomophthoralean Fungus (Pandora Formicae) Shows Molecular Machinery Adjusted for Successful Host Exploitation and Transmission. J. Invertebr. Pathol..

[79] Małagocka J., Jensen A.B., Eilenberg J. (2017). Pandora Formicae, a Specialist Ant Pathogenic Fungus: New Insights into Biology and Taxonomy. J. Invertebr. Pathol..

[80] Boer P. (2008). Observations of Summit Disease in Formica Rufa LINNAEUS, 1761 (Hymenoptera: Formicidae). Myrmecol. News.

[81] Marikovsky P.I. (1962). On Some Features of Behavior of the Ants Formica Rufa L. Infected with Fungous Disease. Ins. Soc..

[82] Hughes D.P., Araújo J.P.M., Loreto R.G., Quevillon L., de Bekker C., Evans H.C. (2016). From So Simple a Beginning: The Evolution of Behavioral Manipulation by Fungi. Adv. Genet..

[83] Soper R.S., Delyzer A.J., Smith L.F.R. (1976). The Genus Massospora Entomopathogenic for Cicadas. Part. II. Biology of Massospora Levispora and Its Host Okanagana Rimosa, with Notes on Massospora Cicadina on the Periodical Cicadas. Ann. Entomol. Soc. Am..

[84] Cooley J.R., Marshall D.C., Hill K.B.R. (2018). A Specialized Fungal Parasite (*Massospora cicadina*) Hijacks the Sexual Signals of Periodical Cicadas (Hemiptera: Cicadidae: Magicicada). Sci. Rep..

[85] Nair K.S.S., McEwen F.L. (1973). Strongwellsea Castrans (Phycomycetes: Entomophthoraceae), a Fungal Parasite of the Adult Cabbage Maggot, Hylemya Brassicae (Diptera: Anthomyiidae). J. Invertebr. Pathol..

[86] Haspel G., Rosenberg L.A., Libersat F. (2003). Direct Injection of Venom by a Predatory Wasp into Cockroach Brain. J. Neurobiol..

[87] Williams F.X. (1942). *Ampulex compressa* (Fabr.), a cockroach-hunting wasp introduced from New Caledonia into Hawaii. Proc. Hawaii. Entomol. Soc..

[88] Libersat F., Delago A., Gal R. (2008). Manipulation of Host Behavior by Parasitic Insects and Insect Parasites. Annu. Rev. Entomol..

[89] Weisel-Eichler A., Libersat F. (2002). Are Monoaminergic Systems Involved in the Lethargy Induced by a Parasitoid Wasp in the Cockroach Prey?. J. Comp. Physiol. A.

[90] Eberhard W.G. (2000). Spider Manipulation by a Wasp Larva. Nature.

[91] Eberhard W.G. (2000). The Natural History and Behavior of Hymenoepimecis Argyraphaga (Hymenoptera: Ichneumonidae) a Parasitoid of Plesiometa Argyra (Araneae: Tetragnathidae). J. Hymenopt. Res..

[92] Eberhard W.G. (2010). Recovery of Spiders from the Effects of Parasitic Wasps: Implications for Fine-Tuned Mechanisms of Manipulation. Anim. Behav..

[93] Eberhard W.G. (2013). The Polysphinctine Wasps Acrotaphus Tibialis, Eruga ca. Gutfreundi, and Hymenoepimecis Tedfordi (Hymenoptera, Ichneumonidae, Pimplinae) Induce Their Host Spiders to Build Modified Webs. Ann. Entomol. Soc. Am..

[94] Thomas F., Schmidt-Rhaesa A., Martin G., Manu C., Durand P., Renaud F. (2002). Do Hairworms (Nematomorpha) Manipulate the Water Seeking Behaviour of Their Terrestrial Hosts?. J. Evol. Biol..

[95] Lafferty K.D., Morris A.K. (1996). Altered Behavior of Parasitized Killifish Increases Susceptibility to Predation by Bird Final Hosts. Ecology.

[96] Martin W.E. (1950). Euhaplorchis calif or niensis n,g., n.sp., Heterophyidae, Trematoda, with notes on its life-cycle. Trans. Am. Microsc. Soc..

[97] Helluy S. (1982). Host-parasite relations of the trematode Microphallus papillorobustus (Rankin, 1940). I. Penetration of cercariae and relationship of the metacercariae with the nervous tissue of Gammarus, intermediate hosts. Ann. Parasitol. Hum. Comp..

[98] Helluy S. (1984). Host-parasite relations of the trematode Microphallus papillorobustus (Rankin 1940). III Factors involved in the behavioral changes of the Gammarus, intermediate hosts and predator tests. Ann. Parasitol. Hum. Comp..

[99] Helluy S., Thomas F. (2003). Effects of Microphallus Papillorobustus (Platyhelminthes: Trematoda) on Serotonergic Immunoreactivity and Neuronal Architecture in the Brain of Gammarus Insensibilis (Crustacea: Amphipoda). Proc. R. Soc. Lond. B Biol. Sci..

[100] Helluy S., Thomas F. (2010). Parasitic Manipulation and Neuroinflammation: Evidence from the System Microphallus Papillorobustus (Trematoda)—Gammarus (Crustacea). Parasit Vectors.

[101] Berdoy M., Webster J.P., Macdonald D.W. (2000). Fatal Attraction in Rats Infected with Toxoplasma Gondii. Proc. R. Soc. Lond. B Biol. Sci..

[102] Martynowicz J., Augusto L., Wek R.C., Boehm S.L., Sullivan W.J. (2019). Guanabenz Reverses a Key Behavioral Change Caused by Latent Toxoplasmosis in Mice by Reducing Neuroinflammation. MBio.

[103] Berenreiterová M., Flegr J., Kuběna A.A., Němec P. (2011). The Distribution of Toxoplasma Gondii Cysts in the Brain of a Mouse with Latent Toxoplasmosis: Implications for the Behavioral Manipulation Hypothesis. PLoS ONE.

[104] Prandovszky E., Gaskell E., Martin H., Dubey J.P., Webster J.P., McConkey G.A. (2011). The Neurotropic Parasite Toxoplasma Gondii Increases Dopamine Metabolism. PLoS ONE.

[105] Stibbs H.H. (1985). Changes in Brain Concentrations of Catecholamines and Indoleamines in Toxoplasma Gondii Infected Mice. Ann. Trop. Med. Parasitol..

[106] Skallová A., Kodym P., Frynta D., Flegr J. (2006). The Role of Dopamine in Toxoplasma-Induced Behavioural Alterations in Mice: An Ethological and Ethopharmacological Study. Parasitology.

[107] Flegr J., Markoš A. (2014). Masterpiece of Epigenetic Engineering—How Toxoplasma Gondii Reprogrammes Host Brains to Change Fear to Sexual Attraction. Mol. Ecol..

[108] Lim A., Kumar V., Dass S.A.H., Vyas A. (2013). Toxoplasma Gondii Infection Enhances Testicular Steroidogenesis in Rats. Mol. Ecol..

[109] Dass S.A.H., Vyas A. (2014). Toxoplasma Gondii Infection Reduces Predator Aversion in Rats through Epigenetic Modulation in the Host Medial Amygdala. Mol. Ecol..

[110] Yong E. Is This Fungus Using a Virus to Control an Animal’s Mind?—The Atlantic. https://www.theatlantic.com/science/archive/2018/07/is-this-fungus-using-a-virus-to-mind-control-an-animal/565982/.

[111] Rossi M., Castiglioni P., Hartley M.-A., Eren R.O., Prével F., Desponds C., Utzschneider D.T., Zehn D., Cusi M.G., Kuhlmann F.M. (2017). Type I Interferons Induced by Endogenous or Exogenous Viral Infections Promote Metastasis and Relapse of Leishmaniasis. Proc. Natl. Acad. Sci. USA.

[112] Adaui V., Lye L.-F., Akopyants N.S., Zimic M., Llanos-Cuentas A., Garcia L., Maes I., De Doncker S., Dobson D.E., Arevalo J. (2016). Association of the Endobiont Double-Stranded RNA Virus LRV1 With Treatment Failure for Human Leishmaniasis Caused by Leishmania Braziliensis in Peru and Bolivia. J. Infect. Dis..

[113] Bourreau E., Ginouves M., Prévot G., Hartley M.-A., Gangneux J.-P., Robert-Gangneux F., Dufour J., Sainte-Marie D., Bertolotti A., Pratlong F. (2016). Presence of Leishmania RNA Virus 1 in Leishmania Guyanensis Increases the Risk of First-Line Treatment Failure and Symptomatic Relapse. J. Infect. Dis..

[114] Grybchuk D., Akopyants N.S., Kostygov A.Y., Konovalovas A., Lye L.-F., Dobson D.E., Zangger H., Fasel N., Butenko A., Frolov A.O. (2018). Viral Discovery and Diversity in Trypanosomatid Protozoa with a Focus on Relatives of the Human Parasite Leishmania. Proc. Natl. Acad. Sci. USA.

[115] Riegler M., O’Neill S.L., Dr M.D.P., Falkow S., Rosenberg E., Schleifer K.-H., Stackebrandt E. (2006). The Genus Wolbachia. The Prokaryotes.

[116] Miller W.J. (2013). Bugs in Transition: The Dynamic World of Wolbachia in Insects. PLoS Genet..

[117] Werren J.H. (1997). Biology of Wolbachia. Annu. Rev. Entomol..

[118] Werren J.H., Baldo L., Clark M.E. (2008). Wolbachia: Master Manipulators of Invertebrate Biology. Nat. Rev. Microbiol..

[119] Albertson R., Casper-Lindley C., Cao J., Tram U., Sullivan W. (2009). Symmetric and Asymmetric Mitotic Segregation Patterns Influence Wolbachia Distribution in Host Somatic Tissue. J. Cell Sci..

[120] Albertson R., Tan V., Leads R.R., Reyes M., Sullivan W., Casper-Lindley C. (2013). Mapping Wolbachia Distributions in the Adult Drosophila Brain. Cell. Microbiol..

[121] Casper-Lindley C., Kimura S., Saxton D.S., Essaw Y., Simpson I., Tan V., Sullivan W. (2011). Rapid Fluorescence-Based Screening for Wolbachia Endosymbionts in Drosophila Germ Line and Somatic Tissues. Appl. Environ. Microbiol..

[122] Strunov A., Schneider D.I., Albertson R., Miller W.J. (2016). Restricted Distribution and Lateralization of Mutualistic Wolbachia in the Drosophila Brain. Cell. Microbiol..

[123] Bi J., Sehgal A., Williams J.A., Wang Y.-F. (2018). Wolbachia Affects Sleep Behavior in Drosophila Melanogaster. J. Insect Physiol..

[124] Evans O., Caragata E.P., McMeniman C.J., Woolfit M., Green D.C., Williams C.R., Franklin C.E., O’Neill S.L., McGraw E.A. (2009). Increased Locomotor Activity and Metabolism of Aedes Aegypti Infected with a Life-Shortening Strain of Wolbachia Pipientis. J. Exp. Biol..

[125] Rohrscheib C.E., Bondy E., Josh P., Riegler M., Eyles D., van Swinderen B., Weible M.W., Brownlie J.C. (2015). Wolbachia Influences the Production of Octopamine and Affects Drosophila Male Aggression. Appl. Environ. Microbiol..

[126] Vale P.F., Jardine M.D. (2015). Sex-Specific Behavioural Symptoms of Viral Gut Infection and Wolbachia in Drosophila Melanogaster. J. Insect Physiol..

[127] Templé N., Richard F.-J. (2015). Intra-Cellular Bacterial Infections Affect Learning and Memory Capacities of an Invertebrate. Front. Zool..

[128] Farahani H.K., Ashouri A., Goldansaz S.H., Shapiro M.S., Pierre J.-S., Baaren J. (2017). van Decrease of Memory Retention in a Parasitic Wasp: An Effect of Host Manipulation by Wolbachia?. Insect Sci..

[129] Peng Y., Nielsen J.E., Cunningham J.P., McGraw E.A. (2008). Wolbachia Infection Alters Olfactory-Cued Locomotion in Drosophila Spp. Appl. Environ. Microbiol..

[130] Peng Y., Wang Y. (2009). Infection of Wolbachia May Improve the Olfactory Response of Drosophila. Chin. Sci. Bull..

[131] Crespigny F.E.C.D., Pitt T.D., Wedell N. (2006). Increased Male Mating Rate in Drosophila Is Associated with Wolbachia Infection. J. Evol. Biol..

[132] Miller W.J., Schneider D. (2012). Endosymbiotic Microbes as Adaptive Manipulators of Arthropod Behavior and Natural Driving Sources of Host Speciation.

[133] Cenit M.C., Sanz Y., Codoñer-Franch P. (2017). Influence of Gut Microbiota on Neuropsychiatric Disorders. World J. Gastroenterol..

[134] Cryan J.F., O’Mahony S.M. (2011). The Microbiome-Gut-Brain Axis: From Bowel to Behavior. Neurogastroenterol. Motil..

[135] Forsythe P., Kunze W.A., Bienenstock J. (2012). On Communication between Gut Microbes and the Brain. Curr. Opin. Gastroenterol..

[136] Shreiner A.B., Kao J.Y., Young V.B. (2015). The Gut Microbiome in Health and in Disease. Curr. Opin. Gastroenterol..

[137] Archie E.A., Theis K.R. (2011). Animal Behaviour Meets Microbial Ecology. Anim. Behav..

[138] Ezenwa V.O., Gerardo N.M., Inouye D.W., Medina M., Xavier J.B. (2012). Animal Behavior and the Microbiome. Science.

[139] Burgener N., Dehnhard M., Hofer H., East M.L. (2009). Does Anal Gland Scent Signal Identity in the Spotted Hyaena?. Anim. Behav..

[140] Theis K.R., Venkataraman A., Dycus J.A., Koonter K.D., Schmitt-Matzen E.N., Wagner A.P., Holekamp K.E., Schmidt T.M. (2013). Symbiotic Bacteria Appear to Mediate Hyena Social Odors. Proc. Natl. Acad. Sci. USA.

[141] Lesperance D.N., Broderick N.A. (2020). Microbiomes as Modulators of Drosophila Melanogaster Homeostasis and Disease. Curr. Opin. Insect Sci..

[142] Masuzzo A., Manière G., Viallat-Lieutaud A., Avazeri É., Zugasti O., Grosjean Y., Kurz C.L., Royet J. (2019). Peptidoglycan-Dependent NF-ΚB Activation in a Small Subset of Brain Octopaminergic Neurons Controls Female Oviposition. eLife.

[143] Masuzzo A., Montanari M., Kurz L., Royet J. (2020). How Bacteria Impact Host Nervous System and Behaviors: Lessons from Flies and Worms. Trends Neurosci..

[144] Schretter C.E., Vielmetter J., Bartos I., Marka Z., Marka S., Argade S., Mazmanian S.K. (2018). A Gut Microbial Factor Modulates Locomotor Behaviour in Drosophila. Nature.

[145] Araújo J.P.M., Hughes D.P., Lovett B., St. Leger R.J. (2016). Chapter One—Diversity of Entomopathogenic Fungi: Which Groups Conquered the Insect Body?. Advances in Genetics.

[146] Lezi E., Zhou T., Koh S., Chuang M., Sharma R., Pujol N., Chisholm A.D., Eroglu C., Matsunami H., Yan D. (2018). An Antimicrobial Peptide and Its Neuronal Receptor Regulate Dendrite Degeneration in Aging and Infection. Neuron.

[147] Cao Y., Chtarbanova S., Petersen A.J., Ganetzky B. (2013). Dnr1 Mutations Cause Neurodegeneration in Drosophila by Activating the Innate Immune Response in the Brain. Proc. Natl. Acad. Sci. USA.

[148] Liu Y., Gordesky-Gold B., Leney-Greene M., Weinbren N.L., Tudor M., Cherry S. (2018). Inflammation-Induced, STING-Dependent Autophagy Restricts Zika Virus Infection in the Drosophila Brain. Cell Host Microbe.

[149] Moore J. (2002). Parasites and the Behavior of Animals.

[150] Libersat F., Kaiser M., Emanuel S. (2018). Mind Control: How Parasites Manipulate Cognitive Functions in Their Insect Hosts. Front. Psychol..

[151] Kato K., Awasaki T., Ito K. (2009). Neuronal Programmed Cell Death Induces Glial Cell Division in the Adult Drosophila Brain. Development.

[152] Katzenberger R.J., Loewen C.A., Wassarman D.R., Petersen A.J., Ganetzky B., Wassarman D.A. (2013). A Drosophila Model of Closed Head Traumatic Brain Injury. Proc. Natl. Acad. Sci. USA.

[153] Katzenberger R.J., Ganetzky B., Wassarman D.A. (2016). Age and Diet Affect Genetically Separable Secondary Injuries That Cause Acute Mortality Following Traumatic Brain Injury in Drosophila. G3 (Bethesda).

[154] Katzenberger R.J., Chtarbanova S., Rimkus S.A., Fischer J.A., Kaur G., Seppala J.M., Swanson L.C., Zajac J.E., Ganetzky B., Wassarman D.A. (2015). Death Following Traumatic Brain Injury in Drosophila Is Associated with Intestinal Barrier Dysfunction. eLife.

[155] Balduf W.V. (1926). The Bionomics of Dinocampus Coccinellæ Schrank. Ann. Entomol. Soc. Am..

[156] Zhan L., Xie Q., Tibbetts R.S. (2015). Opposing Roles of P38 and JNK in a Drosophila Model of TDP-43 Proteinopathy Reveal Oxidative Stress and Innate Immunity as Pathogenic Components of Neurodegeneration. Hum. Mol. Genet..

[157] Shieh S.-Y., Bonini N.M. (2011). Genes and Pathways Affected by CAG-Repeat RNA-Based Toxicity in Drosophila. Hum. Mol. Genet.

[158] Dubey S.K., Tapadia M.G. (2018). Yorkie Regulates Neurodegeneration Through Canonical Pathway and Innate Immune Response. Mol. Neurobiol..

[159] Harris N., Braiser D.J., Dickman D.K., Fetter R.D., Tong A., Davis G.W. (2015). The Innate Immune Receptor PGRP-LC Controls Presynaptic Homeostatic Plasticity. Neuron.

[160] Harris N., Fetter R.D., Brasier D.J., Tong A., Davis G.W. (2018). Molecular Interface of Neuronal Innate Immunity, Synaptic Vesicle Stabilization, and Presynaptic Homeostatic Plasticity. Neuron.

[161] Williams J.A., Sathyanarayanan S., Hendricks J.C., Sehgal A. (2007). Interaction Between Sleep and the Immune Response in Drosophila: A Role for the NFκB Relish. Sleep.

[162] Kuo T.-H., Pike D.H., Beizaeipour Z., Williams J.A. (2010). Sleep Triggered by an Immune Response in Drosophila Is Regulated by the Circadian Clock and Requires the NFkappaB Relish. BMC Neurosci..

[163] Toda H., Williams J.A., Gulledge M., Sehgal A. (2019). A Sleep-Inducing Gene, Nemuri, Links Sleep and Immune Function in Drosophila. Science.

[164] Dissel S., Seugnet L., Thimgan M.S., Silverman N., Angadi V., Thacher P.V., Burnham M.M., Shaw P.J. (2015). Differential Activation of Immune Factors in Neurons and Glia Contribute to Individual Differences in Resilience/Vulnerability to Sleep Disruption. Brain Behav. Immun..

[165] Barajas-Azpeleta R., Wu J., Gill J., Welte R., Seidel C., McKinney S., Dissel S., Si K. (2018). Antimicrobial Peptides Modulate Long-Term Memory. PLoS Genet..

[166] Rivero A. (2006). Nitric Oxide: An Antiparasitic Molecule of Invertebrates. Trends Parasitol..

[167] Jacklet J.W. (1997). Nitric Oxide Signaling in Invertebrates. Invertebr. Neurosci..

[168] Weber A.N.R., Tauszig-Delamasure S., Hoffmann J.A., Lelièvre E., Gascan H., Ray K.P., Morse M.A., Imler J.-L., Gay N.J. (2003). Binding of the Drosophila Cytokine Spätzle to Toll Is Direct and Establishes Signaling. Nat. Immunol..

[169] DeLotto Y., DeLotto R. (1998). Proteolytic Processing of the Drosophila Spätzle Protein by Easter Generates a Dimeric NGF-like Molecule with Ventralising Activity. Mech. Dev..

[170] Sutcliffe B., Forero M.G., Zhu B., Robinson I.M., Hidalgo A. (2013). Neuron-Type Specific Functions of DNT1, DNT2 and Spz at the Drosophila Neuromuscular Junction. PLoS ONE.

[171] Zhu B., Pennack J.A., McQuilton P., Forero M.G., Mizuguchi K., Sutcliffe B., Gu C.-J., Fenton J.C., Hidalgo A. (2008). Drosophila Neurotrophins Reveal a Common Mechanism for Nervous System Formation. PLoS Biol..

[172] Thomas F., Guldner E., Renaud F. (2000). Differential Parasite (Trematoda) Encapsulation in Gammarus Aequicauda (Amphipoda). J. Parasitol..

[173] Shen W., Ganetzky B. (2009). Autophagy Promotes Synapse Development in Drosophila. J. Cell Biol..

[174] McDonald M.J., Rosbash M. (2001). Microarray Analysis and Organization of Circadian Gene Expression in Drosophila. Cell.

[175] Lazzaro B.P., Sceurman B.K., Clark A.G. (2004). Genetic Basis of Natural Variation in D. Melanogaster Antibacterial Immunity. Science.

[176] Li J., Terry E.E., Fejer E., Gamba D., Hartmann N., Logsdon J., Michalski D., Rois L.E., Scuderi M.J., Kunst M. (2017). Achilles Is a Circadian Clock-Controlled Gene That Regulates Immune Function in Drosophila. Brain Behav. Immun..

[177] Hartenstein V. (2011). Morphological Diversity and Development of Glia in Drosophila. Glia.

[178] Ito K., Urban J., Technau G.M. (1995). Distribution, Classification, and Development OfDrosophila Glial Cells in the Late Embryonic and Early Larval Ventral Nerve Cord. Rouxs Arch. Dev. Biol..

[179] Edenfeld G., Stork T., Klämbt C. (2005). Neuron-Glia Interaction in the Insect Nervous System. Curr. Opin. Neurobiol..

[180] Kremer M.C., Jung C., Batelli S., Rubin G.M., Gaul U. (2017). The Glia of the Adult Drosophila Nervous System. Glia.

[181] Limmer S., Weiler A., Volkenhoff A., Babatz F., Klämbt C. (2014). The Drosophila Blood-Brain Barrier: Development and Function of a Glial Endothelium. Front. Neurosci..

[182] Petersen A.J., Rimkus S.A., Wassarman D.A. (2012). ATM Kinase Inhibition in Glial Cells Activates the Innate Immune Response and Causes Neurodegeneration in Drosophila. Proc. Natl. Acad. Sci. USA.

[183] Kounatidis I., Chtarbanova S., Cao Y., Hayne M., Jayanth D., Ganetzky B., Ligoxygakis P. (2017). NF-ΚB Immunity in the Brain Determines Fly Lifespan in Healthy Aging and Age-Related Neurodegeneration. Cell Rep..

[184] Kemp M.W., Massey R.C. (2007). The Use of Insect Models to Study Human Pathogens. Drug Discov. Today Dis. Models.

[185] Scully L.R., Bidochka M.J. (2006). Developing Insect Models for the Study of Current and Emerging Human Pathogens. FEMS Microbiol. Lett..

[186] Loreto R.G., Elliot S.L., Freitas M.L.R., Pereira T.M., Hughes D.P. (2014). Long-Term Disease Dynamics for a Specialized Parasite of Ant Societies: A Field Study. PLoS ONE.

[187] Solá Gracia E., de Bekker C., Hanks E.M., Hughes D.P. (2018). Within the Fortress: A Specialized Parasite Is Not Discriminated against in a Social Insect Society. PLoS ONE.

[188] Stroeymeyt N., Grasse A.V., Crespi A., Mersch D.P., Cremer S., Keller L. (2018). Social Network Plasticity Decreases Disease Transmission in a Eusocial Insect. Science.

[189] Hughes D.P. (2014). On the Origins of Parasite-Extended Phenotypes. Integr. Comp. Biol..

